# Inverse Finite Element Method for Reconstruction of Deformation in the Gantry Structure of Heavy-Duty Machine Tool Using FBG Sensors

**DOI:** 10.3390/s18072173

**Published:** 2018-07-06

**Authors:** Mingyao Liu, Xiong Zhang, Han Song, Shiguang Zhou, Zude Zhou, Weijian Zhou

**Affiliations:** 1 School of Mechanical and Electrical Engineering, Wuhan University of Technology, Wuhan 430070, Hubei, China; myliu@whut.edu.cn (M.L.); songhan@whut.edu.cn (H.S.); 213874@whut.edu.cn (S.Z.); zudezhou@whut.edu.cn (Z.Z.); 255464@whut.edu.cn (W.Z.); 2 Hubei Digital Manufacturing Key Laboratory, Wuhan 430070, Hubei, China

**Keywords:** inverse finite element method, gantry structure, heavy-duty machine tool, reconstruction algorithm, FBG sensors

## Abstract

The deformation of the gantry structure in heavy-duty machine tools is an important factor that affects machining accuracy. In order to realize real-time monitoring of the deformation of the gantry structure, which is statically indeterminate and complex in shape, the reconstruction algorithm based on inverse Finite Element Method (iFEM) is proposed and fiber Bragg grating (FBG) sensors are used to measure strain data. The elements of the gantry structure are divided and the displacement functions of each element are determined. The shape function is obtained by substituting degree of freedoms (DOF) of element nodes into displacement functions. Through a differential method, the relation between strain and DOF of element nodes is established by the strain matrices. Subsequently, the DOF of element nodes are obtained by minimizing an error functional defined as the least-squares error between the analytic strain data and the corresponding experimental strains. Considering coordinate transformation and problem-specific displacement boundary conditions, the total deformation of the gantry structure is obtained. Following this, the experiment was carried out. The deformation simulated by ANSYS was used to replace the experimentally measured deformation and then compared with the deformation reconstructed by iFEM under the same loading condition. The accuracy of iFEM for reconstructing deformation of the gantry structure in heavy-duty machine tools is verified. It provides a new view for improving the machining precision of heavy-duty machine tools.

## 1. Introduction

Real-time monitoring of structural deformations using strain measurements is a vital technology for actuation and control of smart structures, as well as for Structural Health Monitoring (SHM) [1,2,3]. This technology is known as ‘‘shape sensing’’. Some researchers have presented deformed shape monitoring by using strain data measured by strain gauge (SG) or fiber Bragg grating (FBG) [4,5,6]. For example, Jones et al. [7] demonstrated an algorithm which utilized strain information from a set of sixteen FBG sensors mounted on a plate to determine the full deformation field of a cantilever honeycomb plate under arbitrary loading conditions. Kim et al. [8] proposed a method to estimate the bridge deflection using FBG strain sensors. Through the application of classical beam theory, they developed a formula to estimate the continuously deflected profile by using strains measured directly from several points. Using geometric formula, cognition of the structural displacements can also yield a real-time reconstruction of structural strains and stresses, while additionally realizing the application of displacement and stress monitoring or shape- and stress-sensing [9]. 

A key solution to improve the machining precision of heavy-duty machine tools is real-time estimation of the deformed shape of important structures using shape sensing. As the deformation of the gantry structure in heavy-duty machine tools is a main factor that affects machining accuracy, real-time monitoring and compensation of gantry structure deformation is vital to improve the processing accuracy. Many of the aforementioned methods rely on classical beam theory and integration method, which are invalid if the structure has a complex shape, for instance, the gantry structure of heavy-duty machine tools. In order to compensate their shortcomings, the inverse Finite Element Method (iFEM) was proposed. Tessler and Spangler [10] developed an iFEM to reconstruct the deformation of a three-dimensional plate based on the minimization of a least-squares functional that uses the complete set of strain measures. Through theoretical analysis, they proved that the iFEM can reconstruct the displacement field exactly. Farahani et al. [11] made use of iFEM to estimate the blank size and predict the strain distribution in sheet metal forming. They demonstrated that the result obtained by iFEM is in good agreement with commercially available finite element software and experimental results. Kefal et al. [12] published a guide that four-node quadrilateral inverse-shell element, iQS4, is used to monitor displacement and stress of a Panamax containership. Using strains obtained from high-fidelity finite element solutions as the experimentally measured strains, the numerical results confirmed the robustness of the iFEM methodology for monitoring multi-axial deformations and stresses of a Panamax containership floating in beam sea waves. iFEM does not only reconstruct mechanical deformation of a beam and plate but also thermal deformation and composite structures, such as sandwich structures. Cerracchio et al. [13] used iFEM to reconstruct mechanical and thermal deformation of typical composite stiffened structures. They also employed iFEM as a general framework and the Refined Zigzag Theory (RZT) as the underlying plate theory to monitor deformation and stress in laminated composite and sandwich structures [3].

Many of the aforementioned algorithms require a simple shape which can satisfy classical beam theory or use traditional SG to obtain strain data—the kind of data that are either inaccurate or difficult to realize outside a laboratory environment. Fast response or real-time monitoring is important for improving machine precision of heavy-duty machine tools. Due to their attractive characteristics, such as lightweight, small size, electromagnetic interference immunity, long-distance transmission, and electric isolation, FBG have been widely used for measuring strain and temperature data [14,15,16,17,18,19]. In order to satisfy the requirement for real-time monitoring, we used FBG to measure strain data, which is different from the traditional iFEM in this paper.

As the gantry structure of heavy-duty machine tools is statically indeterminate and complex in shape, traditional iFEM which mainly focuses on some simple beam structures fails to meet the requirements of reconstruction. In this paper, we monitor, reconstruct and compensate the deformation of the gantry structure using new shape functions based on iFEM. This paper is structured as follows: Firstly, the gantry structure is divided into elements and displacement functions of these elements are selected. The shape function is obtained by substituting degrees of freedom (DOF) of element nodes into displacement functions. Through a differential method, the relation between strain and DOF of element nodes is established. Secondly, the iFEM displacement solution is obtained by minimizing an error functional defined as the least-squares error between the analytic strain data and the corresponding experimental strains. Using coordinate transformation and problem-specific displacement boundary conditions, the deformation of the gantry structure is obtained. Finally, through comparison of the deformation of the gantry structure reconstructed by iFEM and that which was simulated by ANSYS, a commercially available finite element software, the accuracy, convergence, and robustness of the reconstruction algorithm is verified. The reconstruction algorithm based on iFEM in this paper has the following advantages: (1) it can reconstruct the gantry structure deformation of heavy-duty machine tools without considering the magnitude and form of the force acting on it; (2) it can reconstruct the deformation of a gantry structure which is statically indeterminate and complex in shape; (3) in order to realize real-time monitoring, experimental strain data are measured by FBG which possess many merits such as high sensitivity, anti-electromagnetic interference, strong stability, wavelength division multiplexing and easy installation. The reconstruction method based on iFEM presents a new way for real-time monitoring and compensation of gantry structure deformation which is of great significance to improve the machining precision of heavy-duty machine tools.

In the remainder of the paper, the principles of iFEM are assessed (Section 2). The method and experiment setup are then discussed (Section 3). Finally, to examine the predictive capability of the reconstruction algorithm using iFEM, an experiment was established. The deformation simulated by ANSYS was compared with the deformation reconstructed by iFEM under the same loading condition (Section 4). The conclusions of this research are presented in Section 5.

## 2. Inverse Finite Element Formulation for Gantry Structure

**Figure 1:**
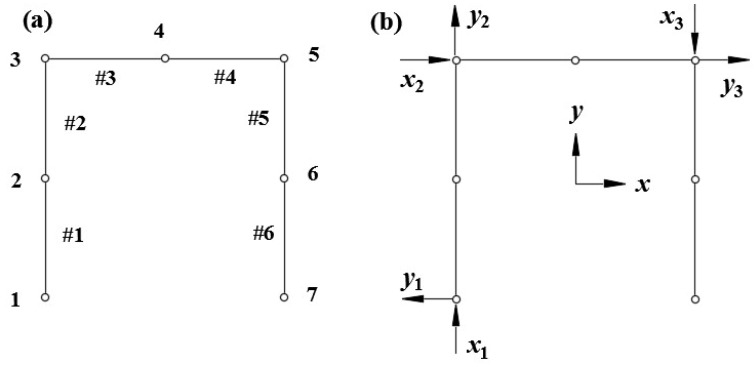
(**a**) The element division of the gantry structure; (**b**) the local and global coordinate systems of the gantry structure.

**Figure 2:**
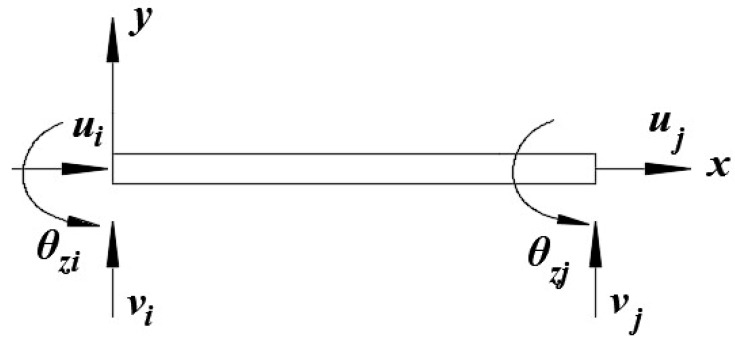
Two-node inverse-beam element.

Firstly, the gantry structure is divided into six elements by element nodes (Figure 1a). In Figure 1a, 1, 2, 3 … 6 denote node numbers and #1, #2, #3 … #6 are element numbers. The result of element division is shown in Figure 1a. Local coordinate systems ($x_{i},y_{i}$) (i=1, 2, 3) are used as each element frame of reference, with its coordinate origin (0, 0) located at one node of the element (Figure 1b). Herein, displacement and strain field of the element are formulated using the local coordinate systems. In order to assemble local element matrices into a global element matrix, suitable transformation matrices defining the local to global transformations can easily be obtained by referring the element nodes to the global coordinates (x, y). This will form a follow-up description.

The two-node inverse-beam element is adopted to express the iFEM formulation for the gantry structure. As shown in Figure 2, it has three displacement DOF per node, the displacement along the x- and y-axes and the rotation about z-axes. It is assumed that the element has a uniform thickness, and that [−h,+h] defines the thickness of the element (Figure 2).

According to the FEM theory, because the number of DOF per element is six, the displacement function along x-axes is selected as a linear polynomial, while the displacement function along y-axes is cubic. They can be expressed as follows:

(1a) $$u=\sum_{i=0}^{1}a_{i}x^{i}$$

(1b) $$v=\sum_{i=0}^{3}a_{i}x^{i}$$

(1c) $$\theta_{z}=\frac{\partial v}{\partial x}$$

where u and v are the displacement of the element along the x- and y-axes and $\theta_{z}$ is the rotation about z-axes. 

Equations (1a)–(1c) are represented as a matrix form:

(2) $$u=\left[\begin{array}{c} u\\ v\\ \theta_{z} \end{array}\right]=\left[\begin{array}{ccc} \begin{array}{c} 1\\ 0\\ 0 \end{array} & \begin{array}{c} x\\ 0\\ 0 \end{array} & \begin{array}{ccc} \begin{array}{c} 0\\ 1\\ 0 \end{array} & \begin{array}{cc} \begin{array}{c} 0\\ x\\ 1 \end{array} & \begin{array}{c} 0\\ x^{2}\\ 2x \end{array} \end{array} & \begin{array}{c} 0\\ x^{3}\\ 3x^{2} \end{array} \end{array} \end{array}\right]\left[\begin{array}{c} a_{0}\\ a_{1}\\ a_{2}\\ a_{3}\\ a_{4}\\ a_{5} \end{array}\right]=\left[M\right]\left[a\right]$$

In order to solve the value of $a_{i}\;\left(i=0,1,2,3\cdots 5\right),$ after substituting the node DOF shown in Figure 2 into Equation (2), it can be described as follows:

(3) $$u_{e}=\left[\begin{array}{c} u_{i}\\ \upsilon_{i}\\ \theta_{zi}\\ u_{j}\\ v_{j}\\ \theta_{zj} \end{array}\right]=\left[\begin{array}{cccccc} 1 & x_{i} & 0 & 0 & 0 & 0\\ 0 & 0 & 1 & x_{i} & x_{i}{}^{2} & x_{i}{}^{3}\\ 0 & 0 & 0 & 1 & 2x_{j} & 3x_{i}{}^{2}\\ 1 & x_{j} & 0 & 0 & 0 & 0\\ 0 & 0 & 1 & x_{j} & x_{j}{}^{2} & x_{j}{}^{3}\\ 0 & 0 & 0 & 1 & 2x_{j} & 3x_{j}{}^{2} \end{array}\right]{\left[\begin{array}{c} a_{0}\\ a_{1}\\ a_{2}\\ a_{3}\\ a_{4}\\ a_{5} \end{array}\right]}_{}=\left[X\right]\left[a\right]$$

where $u_{i}$ $v_{i}$ $\theta_{zi}$ and $u_{j}$ $v_{j}$ $\theta_{zj}$ are the DOF of $i_{st}$ and $j_{st}$ element node.

Replacing [a] with ${\left[X\right]}^{-1}u_{e}$, Equation (2) can be expressed as follows:

(4) $$u=\left[\begin{array}{c} u\\ v\\ \theta_{z} \end{array}\right]=\left[M\right]\left[X\right]{}^{-1}u_{e}=\left[\begin{array}{c} N_{u}\\ N_{w}\\ N_{\theta } \end{array}\right]u_{e}=N*u_{e}$$

where N is the shape function of each element; in order to facilitate the later operation, N is divided into three parts, $\left[\begin{array}{c} u\\ v\\ \theta_{z} \end{array}\right]=\left[\begin{array}{c} N_{u}\\ N_{w}\\ N_{\theta } \end{array}\right]u_{e}$.

**Figure 3:**
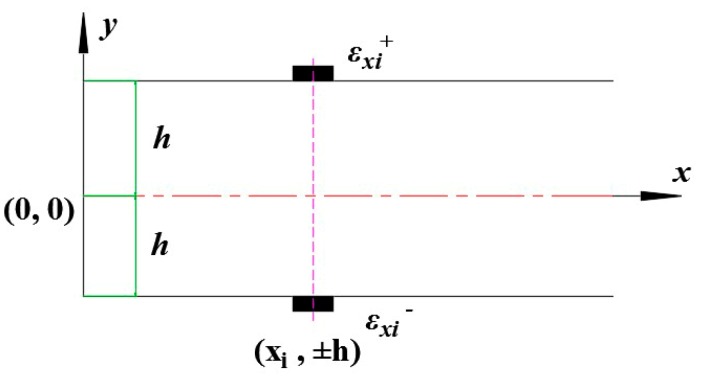
FBG locations within the element at $\left(x_{i},\pm h\right)$

It is crucial for the iFEM technology to obtain strain data from discrete in-situ strain measurements. Due to high sensitivity, anti-electromagnetic interference, strong stability, wavelength division multiplexing, easy installation and other merits, FBG are used to measure strain when the structure is deformed. For the purpose of calculating the membrane and bending strains experimentally, the FBG should be mounted on the top and bottom surfaces of the element as shown in Figure 3.

The experimentally measured membrane strains $\varepsilon_{m}{}^{*}$ and curvatures $\varepsilon_{k}{}^{*}$ can be obtained from the measured strains by FBG at *n* discrete locations $\left(x_{i},\pm h\right)$ (i=1, 2,⋯,n) located within the element. These strains are computed as follows:

(5a) $$\varepsilon_{m}{}^{*}\left(x_{i}\right)=\frac{1}{2}\left(\varepsilon_{x_{i}}{}^{+}+\varepsilon_{x_{i}}{}^{-}\right)$$

(5b) $$\varepsilon_{k}{}^{*}\left(x_{i}\right)=\frac{1}{2h}\left(\varepsilon_{x_{i}}{}^{+}-\varepsilon_{x_{i}}{}^{-}\right)$$

where $\varepsilon_{x_{i}}{}^{+}$ and $\varepsilon_{x_{i}}{}^{-}$ represent the strain measured by FBG on the top and bottom surface locations, respectively.

The relation between displacement and strain data of iFEM are expressed in terms of nodal displacement vector, $u_{e}$, as:

(6a) $$\varepsilon_{m}=\frac{\partial u}{\partial x}=\frac{\partial N_{u}}{\partial x}*u_{e}=B_{m}*u_{e}$$

(6b) $$\varepsilon_{k}=\frac{\partial^{2}w}{\partial x^{2}}=\frac{\partial^{2}N_{w}}{\partial x^{2}}*u_{e}=B_{k}*u_{e}$$

where $B_{m}$ and $B_{k}$ are the strain matrices which contain derivatives of the shape functions, N.

In order to reconstruct the deformation of the element, a least square functional ϕ containing the strains acquired by FBG is proposed. $\varepsilon_{m}{}^{*}$,$\varepsilon_{k}{}^{*}\left(x_{i}\right)$ and $\varepsilon_{m}$, $\varepsilon_{k}$ defined by Equations (5a)–(6b) are minimized with respect to the nodal displacement DOF, $u_{e}$. As strain matrices only depend on the locations of FBG and the length of elements, the unknown DOF of the elements can be obtained. The function ϕ which accounts for the membrane and bending deformations can be written as:

(7) $$\Phi \left(u^{e}\right)=||\varepsilon_{m}\left(u^{e}\right)-\varepsilon_{m}{{}^{*}||}^{2}+||\varepsilon_{k}\left(u^{e}\right)-\varepsilon_{k}{{}^{*}||}^{2}$$

The squared norms expressed in Equation (7) can be written in the form of the normalized Euclidean norms:

(8a) $$||\varepsilon_{m}\left(u^{e}\right)-\varepsilon_{m}{{}^{*}||}^{2}=\frac{1}{n}\overset{n}{\underset{i=1}{\sum }}{\left[\varepsilon_{m}{\left(u^{e}\right)}_{i}-\varepsilon_{m}{}^{*}\left(x_{i}\right)\right]}^{2}$$

(8b) $$||\varepsilon_{k}\left(u^{e}\right)-\varepsilon_{k}{{}^{*}||}^{2}=\frac{{\left(2h\right)}^{2}}{n}\overset{n}{\underset{i=1}{\sum }}{\left[\varepsilon_{k}{\left(u^{e}\right)}_{i}-\varepsilon_{k}{}^{*}\left(x_{i}\right)\right]}^{2}$$

where n is the number of FBG in an element domain and h is the thickness of the element.

Considering each element with a specific length, l, if one of the FBG sensors mounted on the structure surface is invalid, Equations (8a) and (8b) take on the reduced form with the corresponding weighting constant α:

(9a) $$||\varepsilon_{m}{(u^{e})||}^{2}=\alpha \int_{0}^{l}\varepsilon_{m}{\left(u^{e}\right)}^{2}dx$$

(9b) $$||\varepsilon_{k}{\left(u^{e}\right)||}^{2}=\alpha {\left(2h\right)}^{2}\int_{0}^{l}\varepsilon_{k}{\left(u^{e}\right)}^{2}dx$$

where the weight coefficient, α, is set to be small, e.g., $\alpha =10^{-5}$.

Substituting Equations (8a) and (8b) into Equation (7), Equation (7) can be minimized with respect to the nodal displacement DOF, giving rise to:

(10) $$\frac{d\phi }{du^{e}}=k^{e}u^{e}-f^{e}=0$$

The element $k^{e}$ matrix is similar to the stiffness matrix in FEM and only dependent on the locations of FBG and the length of the elements. It can be explicitly written as follows:

(11) $$k^{e}=\frac{1}{n}\sum_{i=1}^{n}B_{m}{\left(x_{i}\right)}^{T}B_{m}\left(x_{i}\right)+\frac{{\left(2h\right)}^{2}}{n}\sum_{i=1}^{n}B_{k}{\left(x_{i}\right)}^{T}B_{k}\left(x_{i}\right)$$

The $f^{e}$, however, is dependent on the measured strain values. It is defined as follows:

(12) $$f^{e}=\frac{1}{n}\sum_{i=1}^{n}B_{m}{\left(x_{i}\right)}^{T}\varepsilon_{m}{}^{*}\left(x_{i}\right)+\frac{{\left(2h\right)}^{2}}{n}\sum_{i=1}^{n}B_{k}{\left(x_{i}\right)}^{T}\varepsilon_{k}{}^{*}\left(x_{i}\right)$$

As displacement and strain field of the element are formulated using the local coordinate systems, the element matrices, $k^{e}$ and $f^{e}$ corresponding to the global coordinate system of the gantry structure can be calculated as:

(13a) $$K=\sum_{e=1}^{nel}{\left(T^{e}\right)}^{T}k^{e}T^{e}$$

(13b) $$F=\sum_{e=1}^{nel}{\left(T^{e}\right)}^{T}f^{e}$$

where $T^{e}$ is the coordinate transformation matrix and nel is the number of elements in the gantry structure.

Accounting for specific problem-dependent displacement boundary conditions, the nodal displacement can be obtained:

(14) $$U_{R}=K_{R}{}^{-1}F_{R}$$

where $K_{R}$ is a positive definite matrix. The solution to these equations for the unknown nodal displacement is efficient because the matrix $K_{R}$ remains unchanged for a determined distribution of FBG, additionally, as the length of the element and its inverse should be calculated only once during the process of the real-time monitoring. $F_{R}$ only depends on the strain data measured by FBG when the structure is deformed, therefore at any strain-measurement update during deformation, Equation (14) provides the solution for the unknown nodal displacement DOF, $U_{R}$.

## 3. Method and Experiment Setup

In this section, operating principles of FBG and the method of the experiment which was carried out to evaluate the applicability of iFEM are discussed. The deformation of the gantry structure contains axial displacement, *u*, and longitudinal displacement, *v*, which cannot be measured synchronously by laser displacement sensors in the laboratory. Therefore, in lieu of the experimentally measured deformation, the deformation simulated by ANSYS, is used to compare with the deformation reconstructed by experimental strain data measured by FBG under the same loading condition. Consequently, the method of strain measurement by FBG, the distribution of FBG on the gantry structure surface and the details of ANSYS simulation are presented.

### 3.1. Operating Principle of an FBG

**Figure 4:**
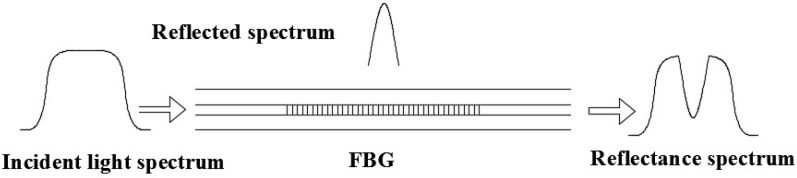
The structure and reflection principle of FBG.

FBG is a periodic distribution of space phase in the core by UV exposure, as shown in Figure 4. When a beam of light is applied to the fiber grating, the periodicity of the refractive index allows only a specific wavelength of light to be reflected. The reflection wavelength satisfies the Bragg scattering condition which can be described as follows:

(15) $$\lambda_{B}=2n\Lambda$$

where n and Λ are effective refractive index of grating and grid period.

The relation between the change in the FBG central wavelength and strain and temperature can be expressed as:

(16) $$\frac{\Delta \lambda_{B}}{\lambda_{B}}=\left(1-P_{e}\right)*\varepsilon +K_{T}*\Delta T$$

where $P_{e}$ is the effective photoelastic coefficient, with the effective photoelastic coefficient of ordinary optical fiber material being 0.22, $K_{T}$ is the temperature sensitivity coefficient, ε and ΔT are the strain data and the change of temperature, respectively.

As the experiment was carried out in a constant temperature environment (ΔT=0), the effect of temperature on the change of FBG central wavelength can be neglected. When the fiber grating is subjected to a pull force, the reflection wavelength of the fiber grating is influenced by the deformation and the change in the refractive index of the optical fiber, caused by the photoelastic effect. The relation between the wavelength change of the fiber grating and strain is:

(17) $$\frac{\Delta \lambda_{B}}{\lambda_{B}}=\left(1-P_{e}\right)*\varepsilon$$

### 3.2. The Distribution of FBG

**Figure 5:**
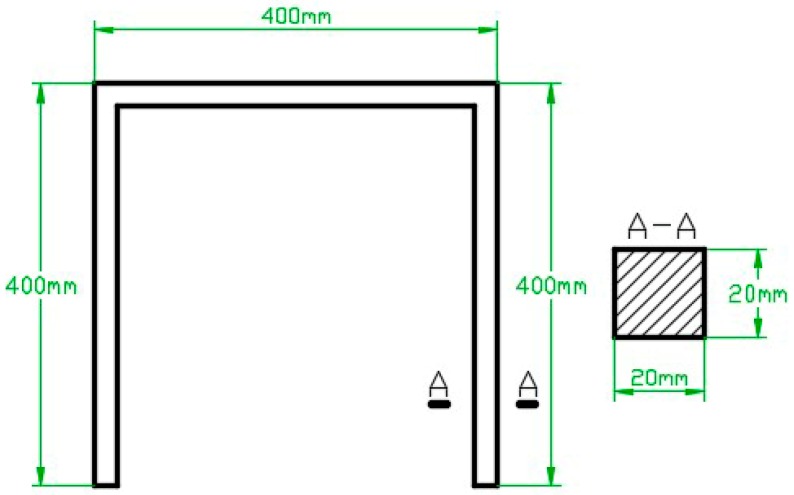
The size parameters of the gantry structure.

In the experiment, the size parameters of the gantry structure are expressed in Figure 5, with Young’s modulus E=210 GPa and Poisson ratio v=0.3. Element division results of the gantry structure are shown in Figure 1a. 

**Figure 6:**
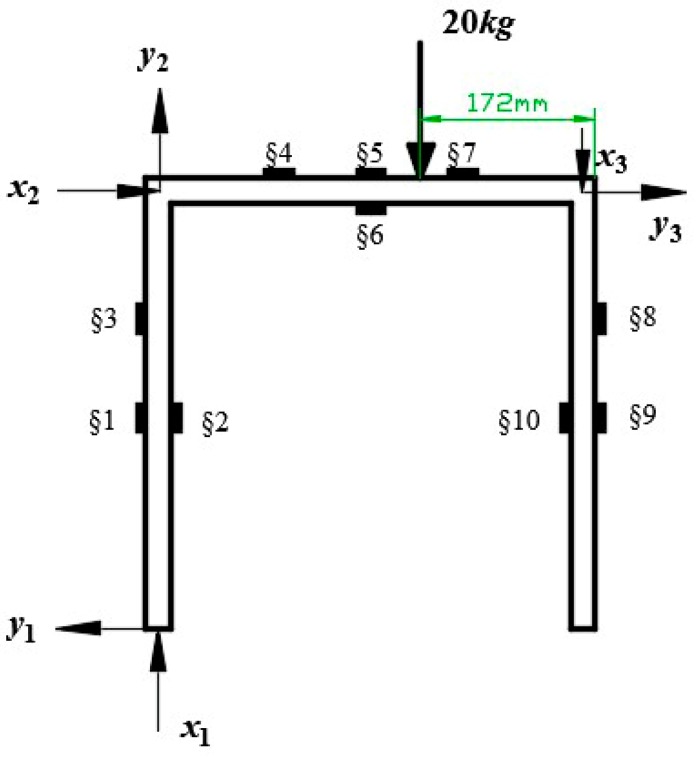
The loading positions and distributions of FBG.

In order to verify the correctness of iFEM when reconstructing the deformation of the gantry structure, as shown in Figure 6, 20 kg weight was loaded on the load block and an ANSYS simulation with the same loading situation was used. As per Section 2, each element was equipped with at least a FBG to measure strain data within the element. It is difficult to satisfy this requirement if the shape of the analyzed structure is complex or consists of a lot of elements. Gherlone et al. [20] developed a conclusion that the bending strain polynomial of the loaded structure is quadratic and the unloaded structure is linear, while the membrane strain remains constant in pure bending conditions. As the gantry structure is only loaded vertically in this experiment, we can obtain strain data at any position of the structure by determining the strain polynomial through discrete FBG mounted on its surface. The plan of FBG distribution is shown in Figure 6 and §1, §2, ⋯, §10 denote the notation of FBG.

The strain data measured by FBG contain membrane and bending strain. Under the premise of establishing the relation of bending strain, $\varepsilon_{s}$ and bending curvature, $\varepsilon_{b}$, with $\varepsilon_{s}=h*\varepsilon_{b}$, the value of measured strain can be expressed:

(18) $$\varepsilon =\varepsilon_{m}+\varepsilon_{s}$$

Because the membrane strain remains constant when the gantry structure is loaded vertically in the experiment, according to Equation (5a), its value at any position can be obtained as follows:

(19) $$\varepsilon_{m}=\frac{\varepsilon^{+}+\varepsilon^{-}}{2}$$

where $\varepsilon^{+}$ and $\varepsilon^{-}$ denote that strain are measured by FBG on the top and bottom surface locations, such as FBG §1 and §2, §5 and §6, or §9 and §10.

According to the conclusion made by Gherlone et al., the bending strain is described as:

(20) $$\varepsilon_{s}=\overset{n}{\underset{i=0}{\sum }}b_{i}x^{i}=\varepsilon -\varepsilon_{m}$$

where the structure is loaded, i=2; the structure is unloaded, i=1. 

Using the strain data measured by FBG, the bending strain polynomial is determined. This can deduce bending strain data at any position on the structure.

By using the above theoretical analysis, the plan of FBG distribution in vertical loading conditions is determined in Figure 6 and strain data at any position can be obtained. The locations of FBG are shown in Table 1.

For illustration purposes only, the above analysis and the plan of FBG distributions are restricted to the structure loaded vertically as shown in Figure 6; naturally, the FBG distribution of other loading conditions may be considered according to practical applications.

### 3.3. The ANSYS Simulation

**Figure 7:**
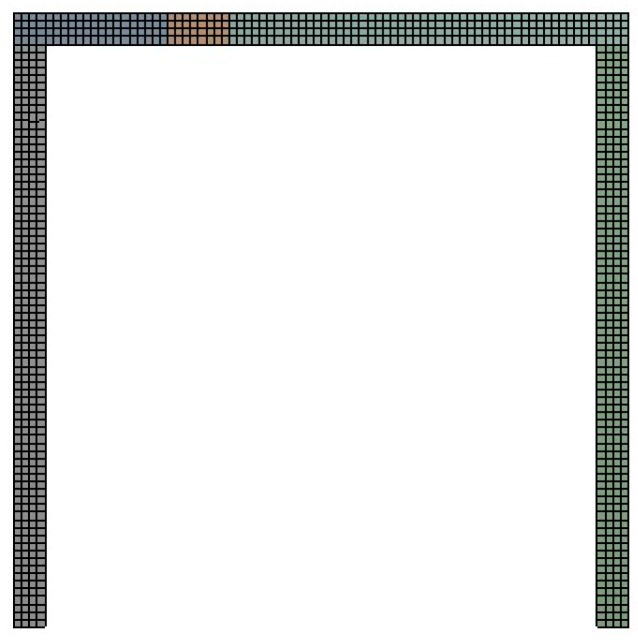
The mesh division result of gantry structure in ANSYS.

In lieu of the experimentally measured deformation, an ANSYS simulation was carried out. The deformation of the gantry structure computed by ANSYS was compared with that which was reconstructed by iFEM. To model the gantry structure, Hex dominant elements were employed. The message of elements and nodes of the ANSYS simulation is shown in Table 2. Due to fine mesh division, and the use of the same loading conditions and boundary conditions as the experimental case, the deformation of the gantry structure computed by ANSYS simulation is quite reliable and the comparison of ANSYS simulation deformation and iFEM deformation is persuasive. Figure 7 shows the result of mesh division.

## 4. Experiment and Analysis

As per the method and setup of the experiment detailed in Section 3, the deformations of the gantry structure reconstructed by iFEM and computed by ANSYS were compared under the same loading condition and boundary condition. 

**Figure 8:**
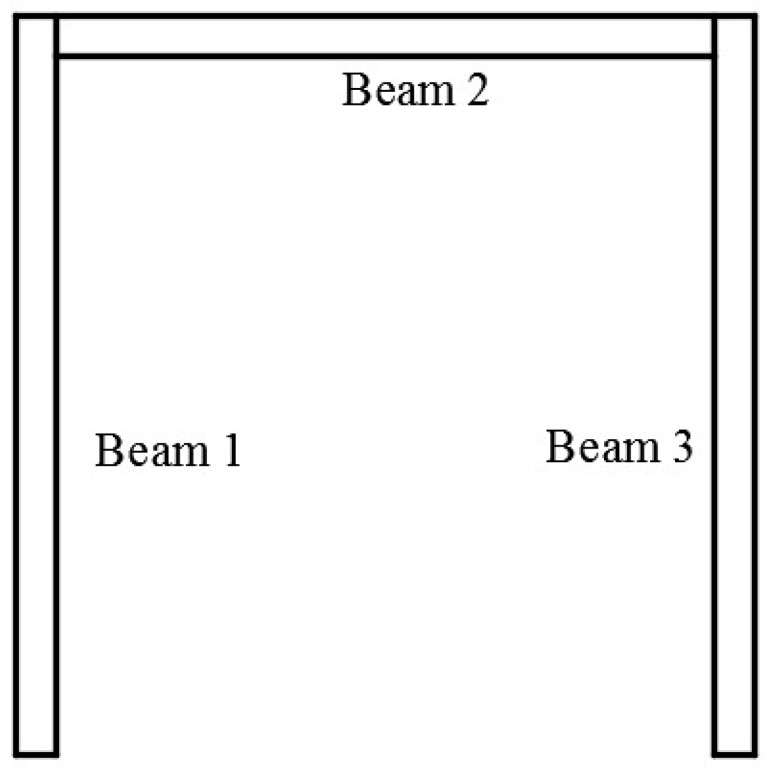
The division result of the gantry structure.

**Figure 9:**
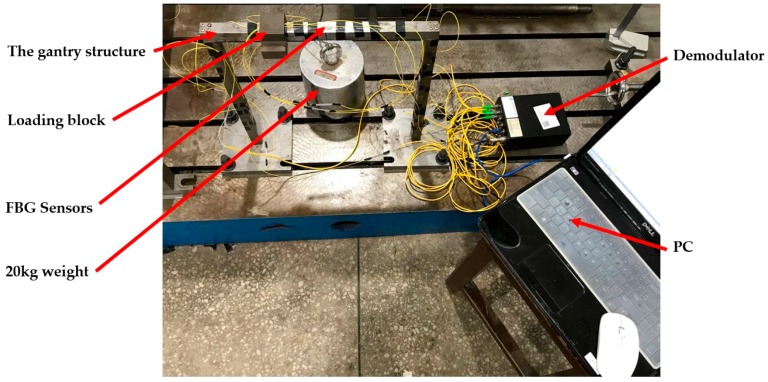
The experimental setup.

For the convenience of displaying the experimental results, the membrane and bending deformation of the gantry structure are formulated using the local coordinate systems and the gantry structure is divided into three beams, shown in Figure 8. Figure 9 shows the experimental setup. As shown in Figure 9, in the experiment, the 20 kg weight was placed on the loading block which ensured that the force was constant along the z-axis of the gantry structure. As the gantry structure has the same constraint in the direction of the z-axis and its material properties are isotropic, the 3-D gantry structure in Figure 9 can be converted to a 2-D structure. When the gantry structure is unloaded, the initial wavelength of FBG is measured and stored into a PC by a demodulator. When the 20 Kg weight is put on the loading block, the wavelength change of FBG can be obtained. Using Equation (17), the strain data can be calculated. Table 3 shows the strain results of FBG.

**Figure 10:**
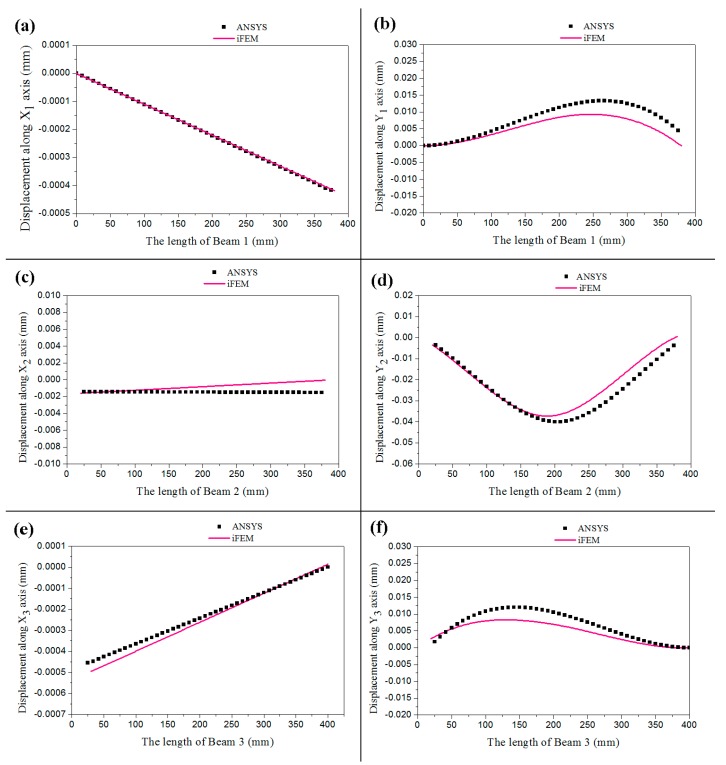
The experimental results. (**a**): The displacement of Beam 1 along x-axis; (**b**): The displacement of Beam 1 along y-axis; (**c**): The displacement of Beam 2 along x-axis; (**d**): The displacement of Beam 2 along y-axis; (**e**): The displacement of Beam 3 along x-axis; (**f**): The displacement of Beam 3 along y-axis.

Figure 10a–f show the comparison result of the membrane and bending displacement in beams 1–3, respectively. The continuous curves indicate the deformation reconstructed by iFEM while the discrete points are the deformation computed by ANSYS in the figures. They show that the deformation of the gantry structure obtained by iFEM coincides with that computed by ANSYS. It certifies that iFEM for reconstruction of deformation in the gantry structure of heavy-duty machine tools is accurate. 

As shown in Figure 10b–e, the largest reconstruction error is 15%, which is at the joints of Beam 1, Beam 2, and Beam 3 since the deformation of the structure is complex and concentration of stress occurs in the joints. These factors will cause difficulties for iFEM to reconstruct deformation and FBG to measure strain. Therefore, the position of FBG should be far away from the joints where the strain changes greatly.

**Figure 11:**
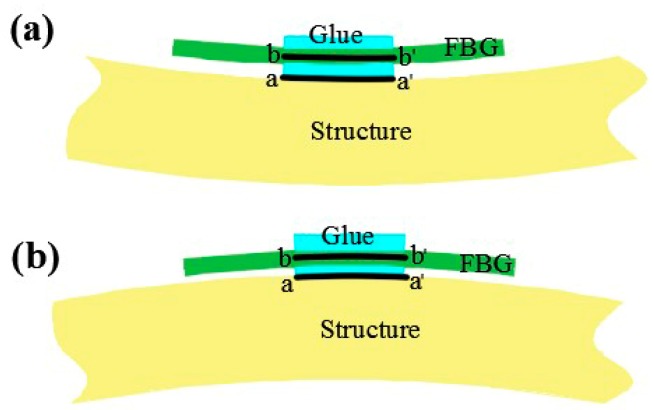
(**a**) The actual measuring position of FBG when the structure is pressed; (**b**) the actual measuring position of FBG when the structure is pulled.

The glue, the black tape and FBG position errors are additional factors which lead to reconstruction error. As the glue and the black tape cannot completely fix the FBG to the surface of the measured structure, there is an error between the strain measured by the FBG and the actual strain. As for FBG position errors, as shown in Figure 11a, when the structure is pressed, due to the thickness of the glue, the measuring position of the FBG is the $b-b^{\prime }$ cross section, while the actual deformation position of the structure is the $a-a^{\prime }$ cross section. The relation of strain data between the two positions can be expressed as follows:

(21) $$\left|\varepsilon_{aa^{\prime }}{}^{-}\right|<\left|\varepsilon_{bb^{\prime }}{}^{-}\right|$$

where $\varepsilon_{aa^{\prime }}{}^{-}$ is the actual strain of the structure when it is pressed, $\varepsilon_{bb^{\prime }}{}^{-}$ is the strain data measured by FBG.

As shown in Figure 11b, when the structure is pulled, the relation of strain data between the two positions is given by

(22) $$\left|\varepsilon_{aa^{\prime }}{}^{+}\right|<\left|\varepsilon_{bb^{\prime }}{}^{+}\right|$$

where $\varepsilon_{aa^{\prime }}{}^{+}$ is the actual strain of the structure when it is pulled, $\varepsilon_{bb^{\prime }}{}^{+}$ is the strain data measured by FBG.

## 5. Conclusions

The deformation of the gantry structure in heavy-duty machine tools is the main factor that affects machining accuracy. As it is statically indeterminate and complex in shape, the reconstruction algorithm based on iFEM is proposed to synchronously monitor and compensate for the deformation of the gantry structure in heavy-duty machine tools. The displacement functions of each element are determined. In order to obtain the shape function, degrees of freedom (DOF) of element nodes are substituted into displacement functions. Through differential methods, the relationship between strain and DOF of element nodes is established by the strain matrices, $B_{m}$ and $B_{k}$. Subsequently, the DOF of element nodes are obtained by minimizing a functional defined as the least-squares error between the analytic strain data and the corresponding experimental strains measured by FBG. Considering coordinate transformation and problem-specific displacement boundary conditions, the total deformation of the gantry structure is obtained.

In order to examine the accuracy of iFEM for reconstructing the deformation of the gantry structure, an experiment was carried out. As the deformation of the gantry structure contains both membrane displacement and bending displacement, which cannot be measured synchronously by laser displacement sensors in the laboratory, the deformation simulated by ANSYS was used to replace the experimentally measured deformation and subsequently compared with the deformation reconstructed by experimental strain data under the same loading condition. 

In accordance with the experimental results, the following conclusions can be obtained: (a) the iFEM methodology can accurately monitor and reconstruct the deformation of the gantry structure in heavy-duty machine tools with the reconstruction error controlled to within 15%. (b) the paste position of FBG should be located far from the joints of the gantry structure. (c) the glue, the black tape and FBG position errors are factors which lead to reconstruction error.

The iFEM provides a theoretical basis for improving the machining precision of heavy-duty machine tools. Future efforts will focus on the real-time reconstruction of the base of heavy-duty machine tools, which is also a main factor that affects machining accuracy.

## Figures and Tables

**Table 1:** The locations of FBG (Unit: mm).

FBG Notation	Orientation of FBG	Local Coordinate System	FBG Notation	Orientation of FBG	Local Coordinate System
§1	(125, 10)	$\left(x_{1},y_{1}\right)$	§6	(200,−10)	$\left(x_{2},y_{2}\right)$
§2	(125, −10)	$\left(x_{1},y_{1}\right)$	§7	(300,10)	$\left(x_{2},y_{2}\right)$
§3	(325, 10)	$\left(x_{1},y_{1}\right)$	§8	(100,10)	$\left(x_{3},y_{3}\right)$
§4	(100, 10)	$\left(x_{2},y_{2}\right)$	§9	(300,10)	$\left(x_{3},y_{3}\right)$
§5	(200, 10)	$\left(x_{2},y_{2}\right)$	§10	(300,−10)	$\left(x_{3},y_{3}\right)$

**Table 2:** The message of elements and nodes of the ANSYS simulation.

Element Type	Total No. of Elements	No. of Nodes
Hex dominant	3728	21,035

**Table 3:** The strain data measured by FBG $\left(\times 10^{-6}\right)$.

§1	§2	§3	§4	§5	§6	§7	§8	§9	§10
−1.6575	−0.8266	0.82766	−21.547	−42.211	47.055	−19.012	0.8266	−1.6553	−2.4766

## References

[1] Papa U., Russo S., Lamboglia A., Del Core G., Iannuzzo G. (2017). Health structure monitoring for the design of an innovative UAS fixed wing through inverse finite element method (iFEM). Aerosp. Sci. Technol..

[2] Kefal A., Mayang J.B., Oterkus E., Yildiz M. (2018). Three dimensional shape and stress monitoring of bulk carriers based on iFEM methodology. Ocean Eng..

[3] Cerracchio P., Gherlone M., Di Sciuva M., Tessler A. (2015). A novel approach for displacement and stress monitoring of sandwich structures based on the inverse Finite Element Method. Compos. Struct..

[4] Gherlone M., Cerracchio P., Mattone M., Di Sciuva M., Tessler A. (2014). An inverse finite element method for beam shape sensing: Theoretical framework and experimental validation. Smart Mater. Struct..

[5] Zhang Y., Yang W. (2016). Simultaneous precision measurement of high temperature and large strain based on twisted FBG considering nonlinearity and uncertainty. Sens. Actuators A Phys..

[6] Papantoniou A., Rigas G., Alexopoulos N.D. (2011). Assessment of the strain monitoring reliability of fiber Bragg grating sensor (FBGs) in advanced composite structures. Compos. Struct..

[7] Jones R.T., Bellemore D.G., Berkoff T.A., Sirkis J.S., Davis M.A., Putnam M.A. (1998). Determination of cantilever plate shapes using wavelength division multiplexed fiber Bragg grating sensors and a least-squares strain-fitting algorithm. Smart Mater. Struct..

[8] Kim N.S., Cho N.S. (2004). Estimating deflection of a simple beam model using fiber optic bragg-grating sensors. Exp. Mech..

[9] Kefal A., Oterkus E. (2016). Displacement and stress monitoring of a chemical tanker based on inverse finite element method. Ocean Eng..

[10] Tessler A., Spangler J.L. (2005). A least-squares variational method for full-field reconstruction of elastic deformations in shear-deformable plates and shells. Comput. Methods Appl. Mech. Eng..

[11] Farahani M.K., Shirin M.B., Assempour A. (2014). Development of an inverse finite element method with an initial guess of linear unfolding. Finite Elem. Anal. Des..

[12] Kefal A., Oterkus E. (2016). Displacement and stress monitoring of a Panamax containership using inverse finite element method. Ocean Eng..

[13] Cerracchio P., Gherlone M., Tessler A. (2015). Real-time displacement monitoring of a composite stiffened panel subjected to mechanical and thermal loads. Meccanica.

[14] Kefal A., Yildiz M. (2017). Modeling of Sensor Placement Strategy for Shape Sensing and Structural Health Monitoring of a Wing-Shaped Sandwich Panel Using Inverse Finite Element Method. Sensors.

[15] Fang L., Chen T., Li R., Liu S. (2016). Application of Embedded Fiber Bragg Grating (FBG) Sensors in Monitoring Health to 3D Printing Structures. IEEE Sens. J..

[16] Tian S., Yang Z., Chen X., Xie Y. (2015). Damage detection based on static strain responses using FBG in a wind turbine blade. Sensors.

[17] Liu Q., Yan J., Pham D.T., Zhou Z., Xu W., Wei Q. (2016). Identification and optimal selection of temperature-sensitive measuring points of thermal error compensation on a heavy-duty machine tool. Int. J. Adv. Manuf. Technol..

[18] Huang J., Zhou Z., Liu M., Zhang E., Chen M., Pham D.T. (2015). Real-time measurement of temperature field in heavy-duty machine tools using fiber Bragg grating sensors and analysis of thermal shift errors. Mechatronics.

[19] Liu M.Y., Zhang Z.J., Zhou Z.D., Peng S., Tan Y.G. (2015). A new method based on Fiber Bragg grating sensor for the milling force measurement. Mechatronics.

[20] Gherlone M., Cerracchio P., Mattone M., Di Sciuva M., Tessler A. (2012). Shape sensing of 3D frame structures using an inverse Finite Element Method. Int. J. Solids Struct..

